# Defective IL-17 production is associated with reduced IL-1β levels in response to *Mycobacterium tuberculosis* in HIV+ children with latent tuberculosis infection

**DOI:** 10.3389/fimmu.2026.1784204

**Published:** 2026-09-09

**Authors:** Kamakshi Prudhula Devalraju, Varalakshmi Mallidi, Abhinav Vankayalapati, Rajesh Kumar Radhakrishnan, Anvesh Kumar Bogam, Saloni Prasad, Sai Vaishnavi Dangatla, Sindhu Joshi, Vinay Kumar Nandicoori, Vijaya Lakshmi Valluri, Ramakrishna Vankayalapati, Venkata Sanjeev Kumar Neela

**Affiliations:** 1 Immunology and Molecular Biology Department, Bhagwan Mahavir Medical Research Centre, Hyderabad, Telangana, India; 2 CSIR-Centre for Cellular and Molecular Biology (CSIR-CCMB), Hyderabad, Telangana, India; 3 School of Medicine, Division of Infectious Diseases, Allergy & Immunology, Saint Louis University, St. Louis, MO, United States; 4 National Institute of Immunology, New Delhi, India

**Keywords:** HIV, LTBI (latent TB infection), CD4+ and CD8+ cells, NK cells, IL-17, IL-1β

## Abstract

**Introduction:**

Children with latent tuberculosis (LTBI) infection are more susceptible to *Mycobacterium tuberculosis* (Mtb) disease than adults with LTBI. HIV infection in children significantly enhances the development of LTBI into active TB due to the immature immune system. However, information about the immune responses to Mtb in HIV+ children with LTBI is limited.

**Methods:**

In this study, we compared the immune cell phenotypes of freshly isolated PBMCs as well as Mtb antigen specific cytokine and chemokine production in HIV-LTBI+ and HIV+LTBI+ children. We also identified the factors responsible for reduced IL-1β production by PBMCs from HIV+LTBI+ children.

**Results:**

We found that compared to HIV-LTBI+ (healthy LTBI+) children, HIV+ children with LTBI have significantly fewer exhausted (LAG3+) NK cells and specific CD8+ cell subsets in freshly isolated PBMCs. We also found that in response to Mtb antigens, PBMCs from HIV+LTBI+ children produced less IL-1β, IL-17, IFN-γ and MCP-1 than PBMCs from HIV-LTBI+ children. Recombinant IL-17 significantly increased the production of IL-1β, MCP-1 and MIP-1α; increased the expansion of CD4+CD45RO+CCR7+CD62L+ T cells; and significantly decreased the number of CD3-CD56+CD158b (KIR2DL) expressing NK cells by CFP-10 and ESAT-6 stimulated PBMCs from HIV+LTBI+ children.

**Discussion:**

We demonstrated that defective IL-17 production is associated with decreased secretion of the protective cytokines IL-1β, MCP-1 and MIP-1α in HIV+LTBI+ children. These findings highlight the need to investigate IL-17 mediated mechanisms to develop interventions for children with concurrent HIV and latent tuberculosis.

## Graphical Abstract

**Figure:**
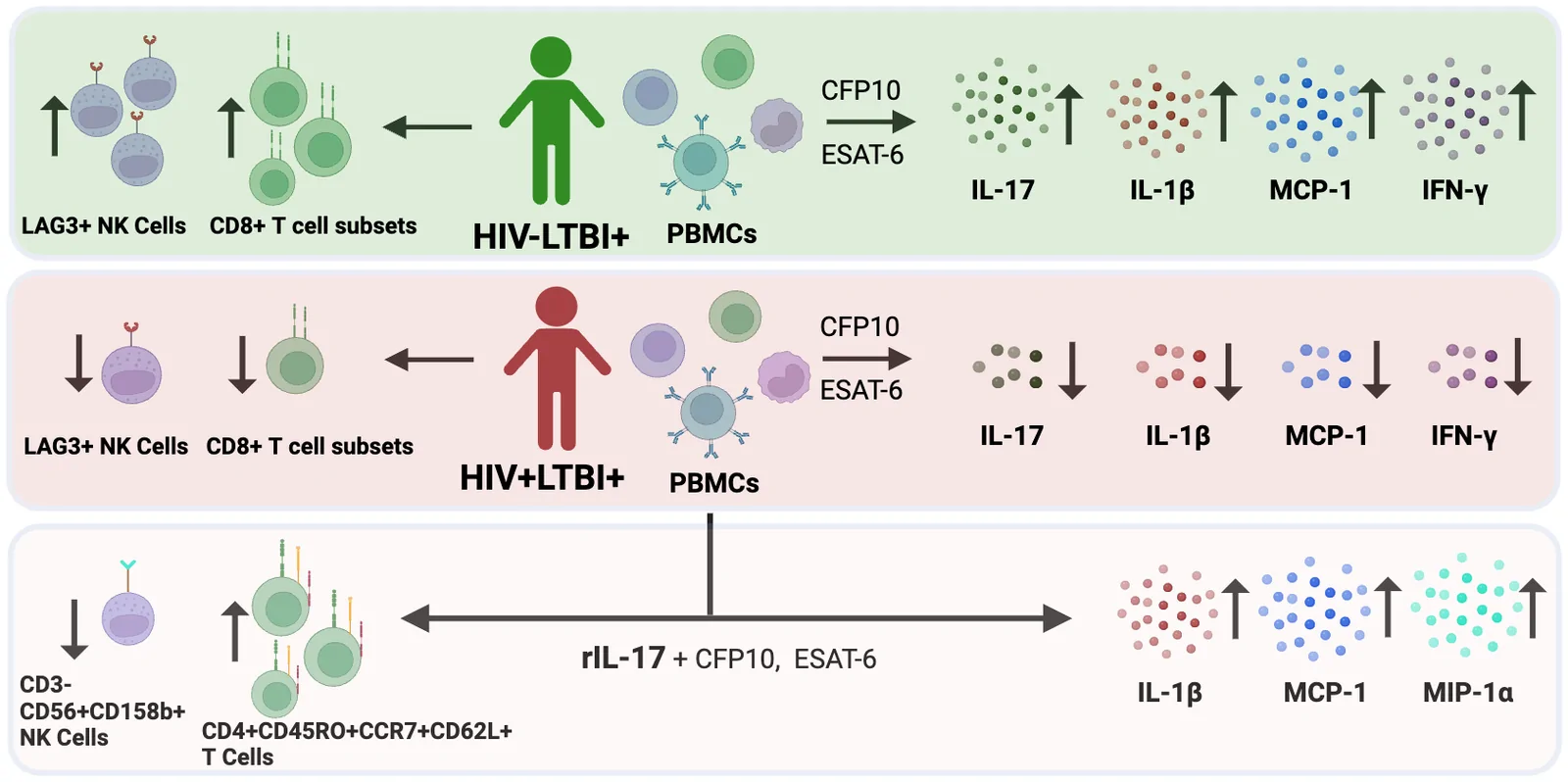
Created in BioRender. Nandicoori, V. (2026) https://BioRender.com/34ttcq5.

## Introduction

*Mycobacterium tuberculosis* (Mtb) infects one-third of the global population and causes almost 1.3 million deaths per year, of which 214,000 occur in children and young adolescents (<15 years) (1, 2). Approximately 90% of people with latent tuberculosis infection (LTBI) have protective immunity and remain free of symptoms, but 10% develop primary tuberculosis (TB) soon after infection with or the reactivation of TB many years later (3). TB is the leading cause of death among HIV-infected people, and more than half a million coinfected people die annually (4). Children are more susceptible to TB infection due to their immature immune systems (5). HIV infection in children markedly increases susceptibility to TB (6). A high risk of TB has been shown in the early stages of HIV disease, even in the presence of normal CD4+ T-cell counts (7). Understanding immune responses to Mtb in children with HIV is important to develop adequate prophylaxis or therapy. The identification of HIV+ children with LTBI who are at greatly increased risk for the development of TB would allow the treatment of only high risk children, facilitating the completion of therapy for LTBI and preventing the future development of TB. Elucidating the nature of the defective immune responses that permit the development of active TB in HIV+LTBI+ pediatric patients is important to identify children at high risk for active TB progression.

The reservoir of CD4+ T cells is reduced among HIV positive children, which is attributable to the virus and the immature immune system (8). In contrast, adults living with HIV have a significant number of memory CD4+ T cells (9). Cytokines such as IFN-γ and TNF-α have been demonstrated to activate macrophages and kill intracellular Mtb, and the production of these cytokines is substantially impaired in HIV infected children compared with adults (10). HIV infected individuals have low T cells numbers required to secrete multiple cytokines which are essential for an effective coordinated immune response against Mtb (11). Additionally, the quantity and cytotoxic function of natural killer (NK) cells are reduced in HIV infected adolescents (12), limiting early innate responses and contributing further to susceptibility to TB infection. While the systemic impairment of T-cell mediated immunity by HIV is well documented, Mthembu et al. (2024) demonstrated that adults with untreated HIV exhibit a clear loss of IL-17A producing CD8+ T cells within the lung mucosa, identifying this deficiency as a primary driver of impaired tuberculosis immunity at the site of infection (13). However, whether similar mucosal or systemic IL-17 defects govern the vulnerability of HIV infected children with LTBI remains poorly understood.

The impact of Mtb coinfection on immune responses during HIV infection has not been fully characterized. Only a few studies have investigated the effect of HIV and LTBI on children receiving antiretroviral therapy (ART) (14). In this study, we identified the immune cell phenotypes in freshly isolated PBMCs from HIV- and HIV+ children with and without LTBI. We also determined Mtb antigen-specific (ESAT-6 and CFP-10) chemokine and cytokine production by the PBMCs of these children. Furthermore, we identified the mechanism by which the production of the protective cytokine IL-1β is reduced in HIV+LTBI+ children.

## Methods

### Study subjects

HIV+ children: Fifty-two children who were seropositive for HIV, diagnosed primarily by COMBAIDS (Arkray health care Pvt. Ltd.) confirmed by VoxPress (Voxtur Bio. Ltd.) and Tredro HIV 1–2 Ab (Meril Diagnostics Pvt. Ltd) kits, were enrolled in the study. Among them, 14 are LTBI+. Children were recruited from Cheyutha, a woman community based organization (WCBO) initiated for individuals infected and affected by HIV/AIDS in Hyderabad, India.

Inclusion and exclusion criteria: Children aged 6 to 17 years with CD4+ cell counts >350 cells/mm^3^ were considered eligible for recruitment. Those with a history of active tuberculosis (ATB), anti-tuberculosis therapy (ATT), other opportunistic infections, chronic illnesses and nutritional deficiencies were considered ineligible. All the children included in the study received anti-retroviral therapy (ART).

Healthy Children: One hundred and fifty healthy HIV seronegative children aged 6 to 17 years were recruited as controls. Among them, 25 are LTBI+. Healthy children had no history of TB or ATT/ART. Those with any other immunosuppressive conditions, opportunistic infections or known nutritional deficiencies were excluded from the study.

To differentiate LTBI from active TB in the study groups, a stringent clinical and diagnostic screening questionnaire was administered. Children with active TB were not included in the study. Active TB is determined by clinician based on presence of signs or symptoms such as persistent cough, hemoptysis, fever, unintended weight loss or failure to thrive, fatigue or lethargy, night sweats, pleuritic chest pain, or other evidence of extra-pulmonary TB. If clinical signs or symptoms of TB are present, CXR or culture were performed to rule out active TB.

**Figure 1:**
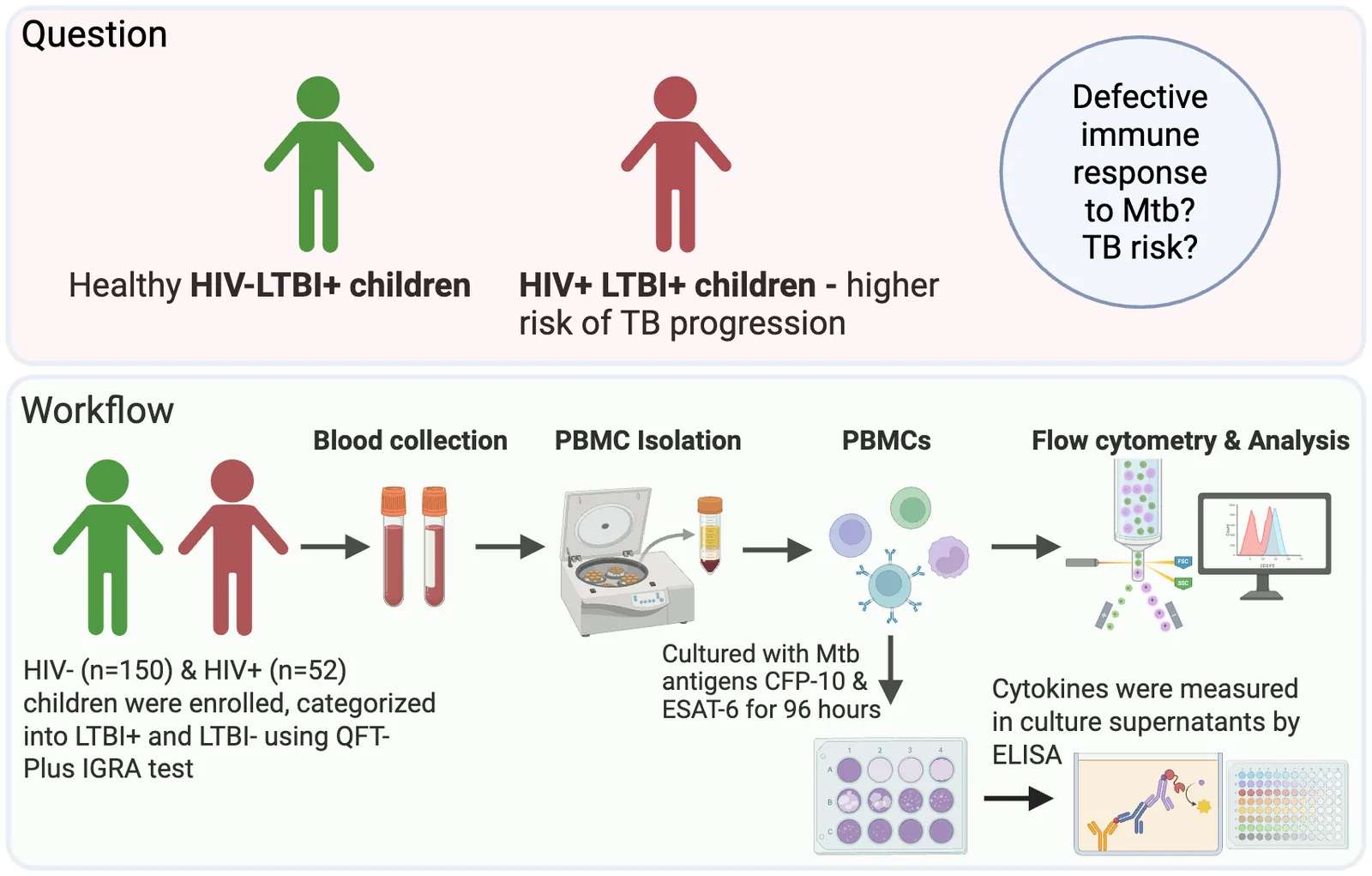
Study outline. A schematic of the study outline. A total of 52 HIV+ and 150 HIV- children in the age range of 6–17 years were prospectively enrolled and categorized into LTBI+ and LTBI- based on interferon gamma release assay. Blood was collected, PBMCs were isolated and immune cell phenotyping was performed using flow cytometry. PBMCs were cultured with Mtb antigens, supplemented with rIL-17 cytokine and antigen specific cytokines/chemokines were measured by ELISA. Analysis was performed as appropriate. Figure created in BioRender. Nandicoori, V. (2026) https://BioRender.com/34ttcq5.

This study was approved by the Institutional Ethics Committee of Bhagwan Mahavir Medical Research Centre, Hyderabad, India. Written and informed assent and consent were obtained from all the enrolled participants their parents/legal guardians. The demographic details of the participants, including age, gender, history of Bacilli-Calmette-Guerin (BCG) vaccination, scar positivity were collected and are shown in **Table 1**. Healthy and HIV+ children were categorized as LTBI- and LTBI+ using the QuantiFERON-TB Gold Plus (QFT-Plus) test and designated HIV+LTBI+, HIV+LTBI- and HIV-LTBI+, HIV-LTBI- respectively. A representative figure outlining the study is shown as **Figure 1**.

**Table 1:** Demographic, anthropometric, and BCG immunization characteristics of the study groups stratified by HIV and latent tuberculosis infection (LTBI) status.

Parameter	HIV Negative (n = 150)		HIV Positive (n = 52)	
	HIV-LTBI-	HIV-LTBI+	HIV+LTBI-	HIV+LTBI+
Total count, n (%)	125 (83.3%)	25 (16.7%)	38 (73.1%)	14 (26.9%)
Age (years)				
Mean ± SD	10.78 ± 3.16	12.88 ± 3.38	12.84 ± 2.97	13.71 ± 3.05
Median (Range)	11.0 (6–17)	13.0 (6–17)	14.0 (6–17)	15.0 (6–17)
Gender, n (%)				
Male	67 (53.6%)	10 (40.0%)	20 (52.6%)	8 (57.1%)
Female	58 (46.4%)	15 (60.0%)	18 (47.4%)	6 (42.9%)
Height (cm)				
Mean ± SD	137.80 ± 21.21	151.00 ± 14.39	133.60 ± 18.20	139.10 ± 17.63
Weight (kg)				
Mean ± SD	31.77 ± 13.59	36.20 ± 11.08	33.92 ± 11.90	35.43 ± 9.97
BMI (kg/m²)				
Mean ± SD	16.48 ± 5.10	15.81 ± 4.15	18.63 ± 4.35	18.15 ± 3.25
BCG Vaccination Status, n (%)				
Yes	122 (97.6%)	23 (92.0%)	38 (100.0%)	14 (100.0%)
No	1 (0.8%)	2 (8.0%)	0 (0.0%)	0 (0.0%)
Unknown	2 (1.6%)	0 (0.0%)	0 (0.0%)	0 (0.0%)
BCG Scar Status, n (%)				
Present	122 (97.6%)	23 (92.0%)	38 (100.0%)	14 (100.0%)
Absent	3 (2.4%)	2 (8.0%)	0 (0.0%)	0 (0.0%)

Values are presented as count (percentage), Mean ± Standard Deviation (SD), or Median (Minimum–Maximum Range).

HIV, Human Immunodeficiency Virus; LTBI, Latent Tuberculosis Infection; BMI, Body Mass Index; BCG, Bacillus Calmette–Guérin; SD, Standard Deviation.

### Determination of latent tuberculosis infection

To assess the LTBI status of the children, a QuantiFERON-TB Gold Plus Interferon-Gamma (IFN-γ) ELISA (QIAGEN, Germany; Cat. No. 622180) was performed according to the manufacturer’s instructions.

### Isolation of PBMCs and culture with the Mtb antigens ESAT-6 and CFP-10

PBMCs from children were isolated by density gradient centrifugation using Ficoll-Hypaque (Serumwerk, Germany; Cat #00524) and cultured according to our published protocols (15, 16).

### Recombinant IL-17 treatment

Recombinant human IL-17A (R&D Systems, USA; Cat# 7955-IL) was reconstituted and diluted in accordance with the manufacturer’s instructions. The recombinant IL-17 at 10 ng/mL concentration was then added to the respective Peripheral Blood Mononuclear cells (PBMCs) well with and without antigens. After 96 hours, the cell-free culture supernatants were collected, aliquoted, and stored at -80 °C until the cytokine concentrations were measured by ELISA. Cultured cells were washed and stained for surface and intracellular markers using flow cytometry as described below.

### Flow cytometry

Antibody pools were prepared for each immune cell subset, T cells, monocytes, and each NK cell subset (as shown in **Supplementary Table 1**), along with appropriate control tubes. 1 × 10^6^ PBMCs per tube were surface stained with the appropriate antibodies and incubated in the dark for 30 minutes to allow surface staining. After incubation, the cells were washed twice with 1X PBS and fixed in 1% paraformaldehyde containing PBS. CD4+CD25+FoxP3+ cells were identified according to our previously published methods (15) using a kit obtained from BioLegend, San Diego, CA, USA. The samples were acquired on a BD FACS Canto II flow cytometer BD Biosciences, San Jose, CA, USA and the data was analyzed using Flowjo v11 software (BD Life Sciences).

### Gating strategies

CD4+ and CD8+ cells were identified after gating on the whole lymphocyte population. T regulatory (Treg) cells were gated as CD4+CD25+FoxP3+ cells. NK cells were gated as CD56+ cells in the CD3- population. CD16-expressing CD56+ cells were identified as double-positive CD16+CD56+ cells. CD16-expressing monocytes were identified as CD14+CD16+ cells. All successive subpopulations were gated as part of the parent population (**Supplementary Figure 1**).

### Antibodies and other reagents

To stain for various immune cell subsets, we used antibodies labeled with fluorochrome tags, as shown in the **Supplementary Table 1**. All antibodies used were obtained from BD Biosciences, San Jose, CA, USA and BioLegend, San Diego, CA, USA.

### Cytokine and chemokine levels were measured by ELISA

ESAT-6– and CFP-10–stimulated PBMC culture supernatants were stored at –80 °C until the cytokine concentrations were measured. In culture supernatants, the levels of the following 8 cytokines and chemokines were measured using ELISA kits as per the manufacturer’s instructions: IL-1α, IFN-γ, IL-1β, IL-6, IL-10, IL-17A, IL-22 and MCP-1 (BioLegend, San Diego, CA, USA). The assay was performed in batches by an independent reader blinded to the specimen status. IL-1β, IL-17, MCP-1 cytokine levels were also measured by ELISA in mitogen stimulated supernatants from the QuantiFERON-TB Gold Plus assay tubes.

### Lactate dehydrogenase cytotoxicity assay

Cytotoxicity in PBMC cultures was determined by measuring lactate dehydrogenase (LDH) release into the culture medium using the CyQUANT™ LDH Cytotoxicity Assay Kit (Invitrogen, Catalog #C20300), following the manufacturer’s protocol. Control and CFP10+ESAT6 antigens stimulated PBMC culture supernatants were collected after 96 hours. LDH activity was quantified spectrophotometrically, and cytotoxicity was expressed as a percentage relative to maximum lysis controls.

### Statistical analysis

Statistical analyses were performed using Prism 10.6.1 software (GraphPad Software, San Diego, CA). To eliminate baseline age and BMI as confounding variables prior to downstream functional assays, antigen specific cytokine profiling, multicolor flow cytometry, and recombinant cytokine supplementation experiments were conducted on age, gender and BMI matched group of individuals across the study groups. Comparative statistics confirmed no significant baseline disparities across this matched subset (p > 0.05), preventing the need for secondary covariate adjustment. Consequently, multi-group data comparisons across the four study groups were evaluated using one-way ANOVA followed by Tukey’s multiple comparison test. All results are expressed as the mean ± SE, with p-values < 0.05 defined as statistically significant.

## Results

### Demographic characteristics of healthy and HIV+ children

As shown in **Table 1**, the study consisted of 150 HIV- children and 52 HIV+ children (a total of 202 participants). The age of HIV- children ranged from 6 to 17 years, similar to that of HIV+ children (6 to 17 years). Among the HIV- children, 125 (83.3%) were LTBI- and 25 (16.7%) were LTBI +. HIV+ children had 38 LTBI- (73.08%) and 14 LTBI+ (26.9%). The HIV- children had mean ages 10.78 ± 3.16 years (LTBI- group), 12.88 ± 3.38 years (LTBI+ group). HIV+ children had mean ages 12.84 ± 2.97 years (LTBI- group), 13.71 ± 3.05 years (LTBI+ group). In the HIV- children males constituted 53.6% (LTBI- group), 40.0% (LTBI+ group) and females were 46.4% (LTBI- group), 60.0% (LTBI+ group). In the HIV+ children males constituted 52.6% (LTBI- group), 57.1% (LTBI+ group) and females were 47.4%(LTBI- group), 42.9% (LTBI+ group).

The HIV- children had a mean BMI of 16.48 ± 5.10 kg/m² (LTBI- group), 15.81 ± 4.15 kg/m² (LTBI+ group); whereas HIV+ children had a mean BMI 18.63 ± 4.35 kg/m² (LTBI- group), 18.15 ± 3.25 kg/m² (LTBI+ group). The clinical profile of HIV+ children, stratified by LTBI status is presented in **Table 2**.

**Table 2:** Clinical and immunological profiles of the HIV positive cohort stratified by LTBI status.

Parameter	Summary Statistic	LTBI Negative (n = 38)	LTBI Positive (n = 14)	Total HIV Cohort (n = 52)
ART Duration (years)	Mean ± SD	9.50 ± 3.56	9.50 ± 4.27	9.44 ± 3.72
	Median (Range)	9 (3 – 16)	10.0 (4 – 15)	9.0 (3 – 16)
Mode of Transmission	Mother-to-Child, n (%)	37 (97.4%)	14 (100.0%)	51 (98.1%)
	Other/Unknown, n (%)	1 (2.6%)	0 (0.0%)	1 (1.9%)
Treatment Patterns	TLD-Based Regimen, n (%)	30 (78.9%)	11 (78.6%)	41 (78.8%)
	ALD-Based Regimen, n (%)	8 (21.1%)	3 (21.4%)	11 (21.2%)
CD4 Count (cells/μL)	Mean ± SD	908.90 ± 434.60	863.40 ± 351.70	896.60 ± 411.10
	Median (Range)	823 (351 – 1998)	785 (415 – 1751)	792 (351 – 1998)
Viral Load Status	Suppressed (TND), n (%)	30 (78.9%)	12 (85.7%)	42 (80.8%)
	Detectable, n (%)	8 (21.1%)	2 (14.3%)	10 (19.2%)

HIV, Human Immunodeficiency Virus; LTBI, Latent Tuberculosis Infection; SD, Standard Deviation; ART, Antiretroviral Therapy; TLD, Tenofovir/Lamivudine/Dolutegravir regimen; ALD, Alternative Abacavir, Lamivudine, and Dolutegravir regimen; TND, Target Not Detected; SD, Standard Deviation. Data are presented as mean ± standard deviation, median (range), or absolute frequency (n) and percentage (%) as indicated. None of the parameters exhibit a statistically significant difference between the two groups (p > 0.05 across all metrics).

### NK cell and T cell phenotypes in freshly isolated PBMCs

**Figure 2:**
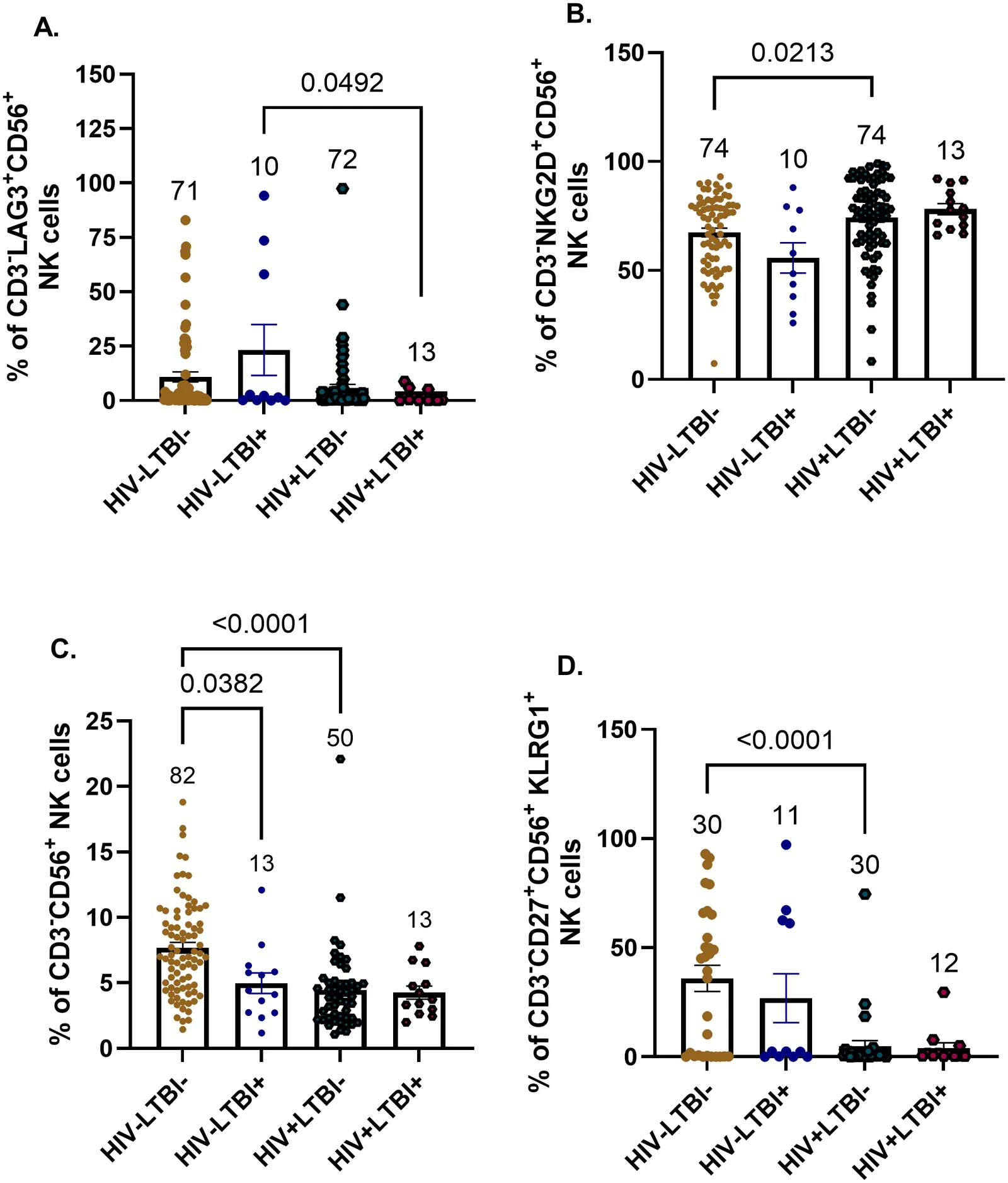
NK cell subpopulations in the freshly isolated PBMC. PBMCs were isolated from HIV-LTBI-, HIV-LTBI+, HIV+LTBI- and HIV+LTBI+ children and stained with antibodies to various NK cell subsets. Percentages of **(A)** CD3-LAG3+CD56+ **(B)** CD3-NKG2D+CD56+ **(C)** CD3-CD56+ **(D)** CD3-CD27+CD56+KLRG1 cells were determined by flow cytometry. The number of donors per group are shown above each bar. *P* values were determined using one-way ANOVA with Tukey’s multiple-comparison test. Mean values, SEM, and *P* values are shown.

**Figure 3:**
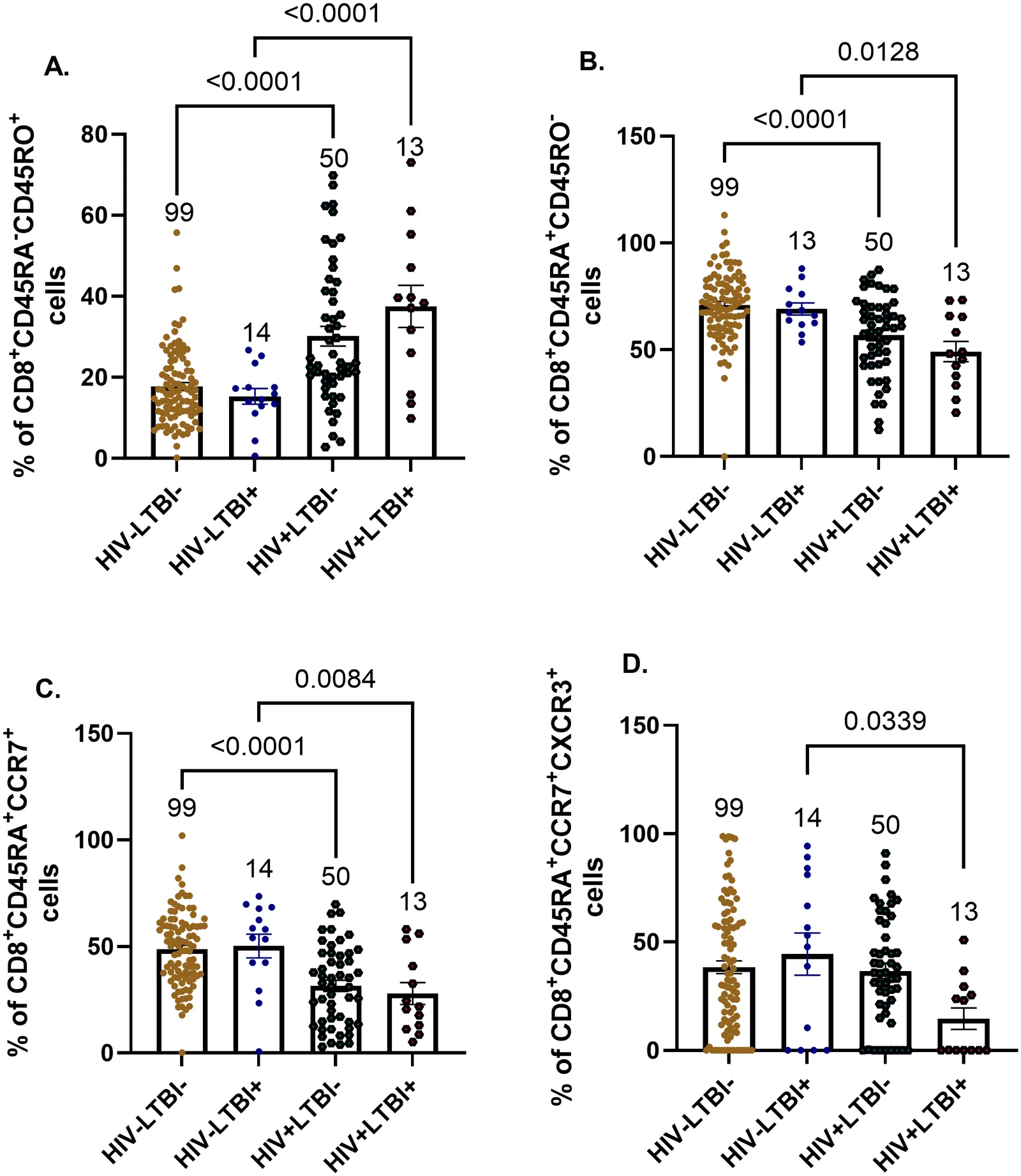
CD8+T cell subpopulations in the freshly isolated PBMC. PBMCs were isolated from HIV-LTBI-, HIV-LTBI+, HIV+LTBI- and HIV+LTBI+ children and were stained with antibodies to T cell subsets. Percentages of **(A)** CD8+CD45RA-CD45RO+ **(B)** CD8+CD45RA+CD45RO- **(C)** CD8+CD45RA+CCR7+ **(D)** CD8+CD45RA+CCR7+CXCR3+ cells were determined by flow cytometry. The number of donors per group are shown above each bar. *P* values were determined using one-way ANOVA with Tukey’s multiple-comparison test. Mean values, SEM, and *P* values are shown.

We compared various immune cell populations among the PBMCs of HIV-LTBI-, HIV-LTBI+, HIV+LTBI-, and HIV+LTBI+ children. The PBMCs of HIV+LTBI+ (HIV+ latent TB) children contained significantly fewer CD3-LAG3+CD56+ NK cells (*P* = 0.0492, **Figure 2A**), CD8+CD45RA+CD45RO- (*P* = 0.0128, **Figure 3B**), CD8+CD45RA+CCR7+ (*P* = 0.0084, **Figure 3C**), CD8+CD45RA+CCR7+ CXCR3+ (*P* = 0.0339, **Figure 3D**) compared to the HIV-LTBI+ (healthy latent TB) children. In contrast, CD8+CD45RA-CD45RO+ (*P* < 0.0001, **Figure 3A**) cells were higher in HIV+LTBI+ children compared to HIV-LTBI+ children.

There were significantly higher number of CD14+CD16+ (*P* = 0.0158, **Supplementary Figure 2A**), CD3-NKG2D+CD56+ NK (*P* = 0.0213, **Figure 2B**), CD8+CD45RA-CD45RO+ cells (*P* < 0.0001, **Figure 3A**), CD8+CD45RA+CD45RO+ cells (*P* = 0.0199, **Supplementary Figure 2B**), CD8+CD45RA+CCR7+CD69+ (*P* = 0.0105, **Supplementary Figure 2C**), CD4+CD25+FoxP3+PD1+ cells (*P* < 0.0001, **Supplementary Figure 2D**) cells in the PBMC of HIV+LTBI- children when compared to the HIV-LTBI- children.

There were significantly less number of CD14-CD16+ (*P* = 0.0008, **Supplementary Figure 3A**), CD16+CD56+ (*P* = 0.0136, **Supplementary Figure 3B**), CD3-CD56+ (*P* < 0.0001, **Figure 2C**), CD3-CD27+CD56+KLRG1+ (*P* < 0.0001, **Figure 2D**), CD3-CD27+CD56+IL21R+ (*P* = 0.029, **Supplementary Figure 3C**), CD4+ T (*P* < 0.0001, **Supplementary Figure 3D**), CD8+CD45RA+CD45RO- (*P* < 0.0001, **Figure 3B**), CD8+CD45RA+CCR7+ (*P* < 0.0001, **Figure 3C**) cells and CD4+CD25+FoxP3+CXCR5+ cells (*P* = 0.0372, **Supplementary Figure 3E**), CD4+CD25+FoxP3+HLADR+ cells (*P* = 0.0054, **Supplementary Figure 3F**) in the PBMCs of HIV+LTBI- children when compared to the HIV-LTBI- children.

### Cytokine and chemokine production by Mtb antigen-stimulated PBMCs

**Figure 4:**
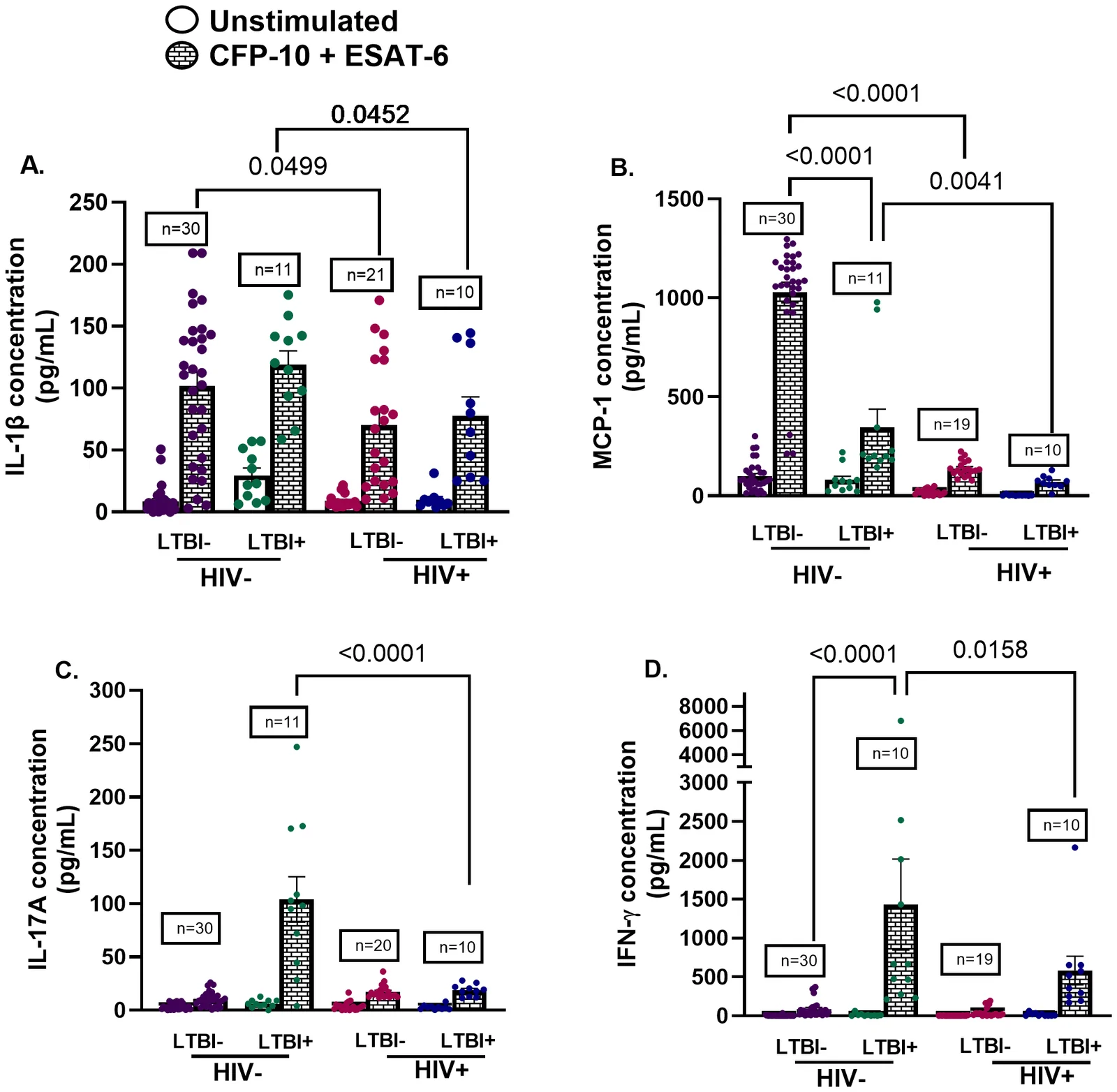
Cytokine and chemokine profiles of ESAT-6 and CFP-10 stimulated PBMC. PBMCs were isolated from HIV-LTBI-, HIV-LTBI+, HIV+LTBI- and HIV+LTBI+ children and cultured with or without ESAT-6 and CFP-10 (10 μg/mL each), as described in methods section. After 96 hours, culture supernatants were collected, and levels of following chemokines and cytokines were measured by ELISA. **(A)** IL-1β **(B)** MCP-1 **(C)** IL-17A **(D)** IFN-γ. The number of donors per group are shown above each bar. *P* values were determined using one-way ANOVA with Tukey’s multiple-comparison test. Mean values, SEM, and *P* values are shown.

We assessed cytokine and chemokine production by freshly isolated PBMCs from HIV-LTBI-, HIV-LTBI+, HIV+LTBI-, and HIV+LTBI+ children. PBMCs were stimulated for 96 hours with Mtb antigens CFP-10 and ESAT-6 (10 µg/mL each) or left unstimulated. ELISA was used to quantify their cytokine and chemokine levels. HIV+LTBI+ children PBMCs produced less IL-1β (*P* = 0.0452, **Figure 4A**), MCP-1 (*P* = 0.0041, **Figure 4B**), IL-17A (*P* < 0.0001, **Figure 4C**) and IFN-γ (*P* = 0.0158, **Figure 4D**) compared to PBMCs of HIV-LTBI+ children after Mtb antigen stimulation. Similarly, HIV+LTBI- children produced low IL-1β (*P* = 0.0499, **Figure 4A**), MCP-1 (*P* < 0.0001, **Figure 4B**), IL-10 (*P* < 0.0001, **Supplementary Figure 4A**), and IL-1α (*P* < 0.0001, **Supplementary Figure 4B**) compared to PBMCs of HIV-LTBI- children after Mtb antigen stimulation. IL-6 (**Supplementary Figure 4C**) and IL-22 (**Supplementary Figure 4D**) production by PBMCs in response to Mtb antigen stimulation did not significantly differ among the four groups.

To assess if HIV+ children are unresponsive to mitogen stimulation, cytokines IL-1β, IL-17, and MCP-1 were measured in supernatants obtained from unstimulated and mitogen (phytohemagglutinin (PHA)) stimulated tubes of the QuantiFERON-TB Gold Plus kit across all four study groups. While IL-1β and IL-17 concentrations were comparable across groups, mitogen stimulated MCP-1 was significantly higher in HIV+LTBI+ children (1453 ± 13.42 pg/mL; Mean ± SEM) than in HIV-LTBI+ controls (1203 ± 19.03 pg/mL; p=0.0247). However, because unstimulated (nil) or baseline MCP-1 levels in both HIV+LTBI+ (1414 ± 22.86 pg/mL) and HIV-LTBI+ (1109 ± 109.5 pg/mL) groups closely matched their respective mitogen stimulated responses, the background subtracted net cytokine response was non-significant. Therefore, MCP-1 was excluded from downstream analysis (**Supplementary Figures 5A-C** and **Supplementary Table 2**).

Pro IL-1β is released upon cell death in the culture supernatants (17). We measured cell death in the above cultured cells using LDH cytotoxicity assay as mentioned in methods section. There are no significant differences in cytotoxicity between groups (p > 0.05) (**Supplementary Figure 6**). Our findings suggest IL-1β detected in culture supernatants represents mature, secreted cytokine rather than the cytosolic pro-IL-1β precursor (**Supplementary Figure 5A**).

### IL-17A increases IL-1β production by Mtb antigen-stimulated PBMCs from HIV+LTBI+ children

**Figure 5:**
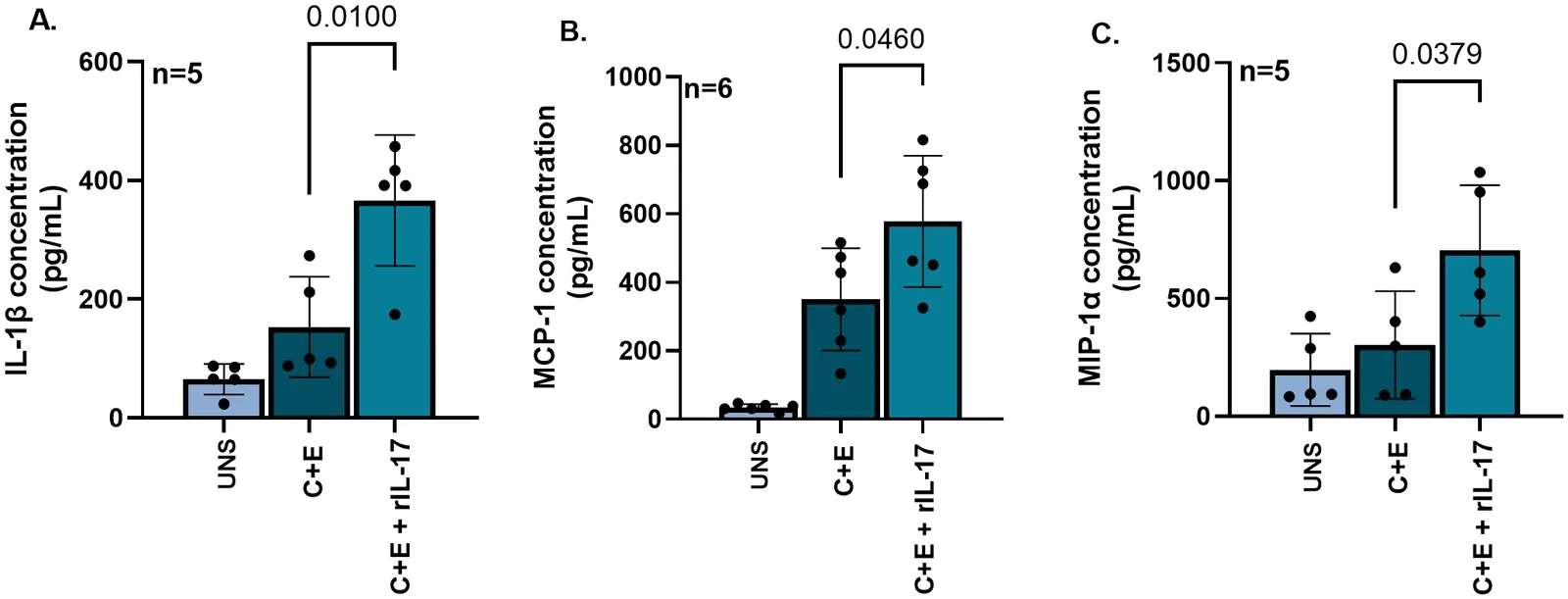
Cytokine and chemokine production by CFP-10 and ESAT-6 stimulated PBMC of HIV+LTBI+ children in the presence or absence of recombinant IL-17. PBMCs were isolated from HIV+LTBI+ children and cultured with or without ESAT-6 and CFP-10 (10 μg/mL each). Some ESAT-6 and CFP-10 (10 μg/mL each) cells were cultured with recombinant IL-17 (10 ng/mL). After 96 hours, culture supernatants were collected, and levels of following chemokines and cytokines were measured by ELISA. **(A)** IL-1β **(B)** MCP-1 **(C)** MIP-1α. The number of donors are shown adjacent to Y-axis. *P* values were determined using one-way ANOVA with Tukey’s multiple-comparison test. Mean values, SEM, and *P* values are shown.

Cytokines produced by NK cells and T cells can enhance antigen-presenting cell function and cytokine production (18). IL-1β is essential for inhibiting Mtb growth (19). We investigated whether recombinant IL-17 enhances IL-1β production by Mtb antigen-stimulated PBMCs. PBMCs from HIV+LTBI+ children were stimulated with ESAT-6 and CFP-10, and some stimulated cells were cultured with recombinant IL-17 (10 ng/mL), as described in the methods section. Recombinant IL-17 significantly increased IL-1β (*P* = 0.01, **Figure 5A**), MCP-1 (*P* = 0.046, **Figure 5B**), and MIP-1α (*P* = 0.0379, **Figure 5C**) production by ESAT-6 and CFP-10 stimulated HIV+LTBI+ children PBMC.

**Figure 6:**
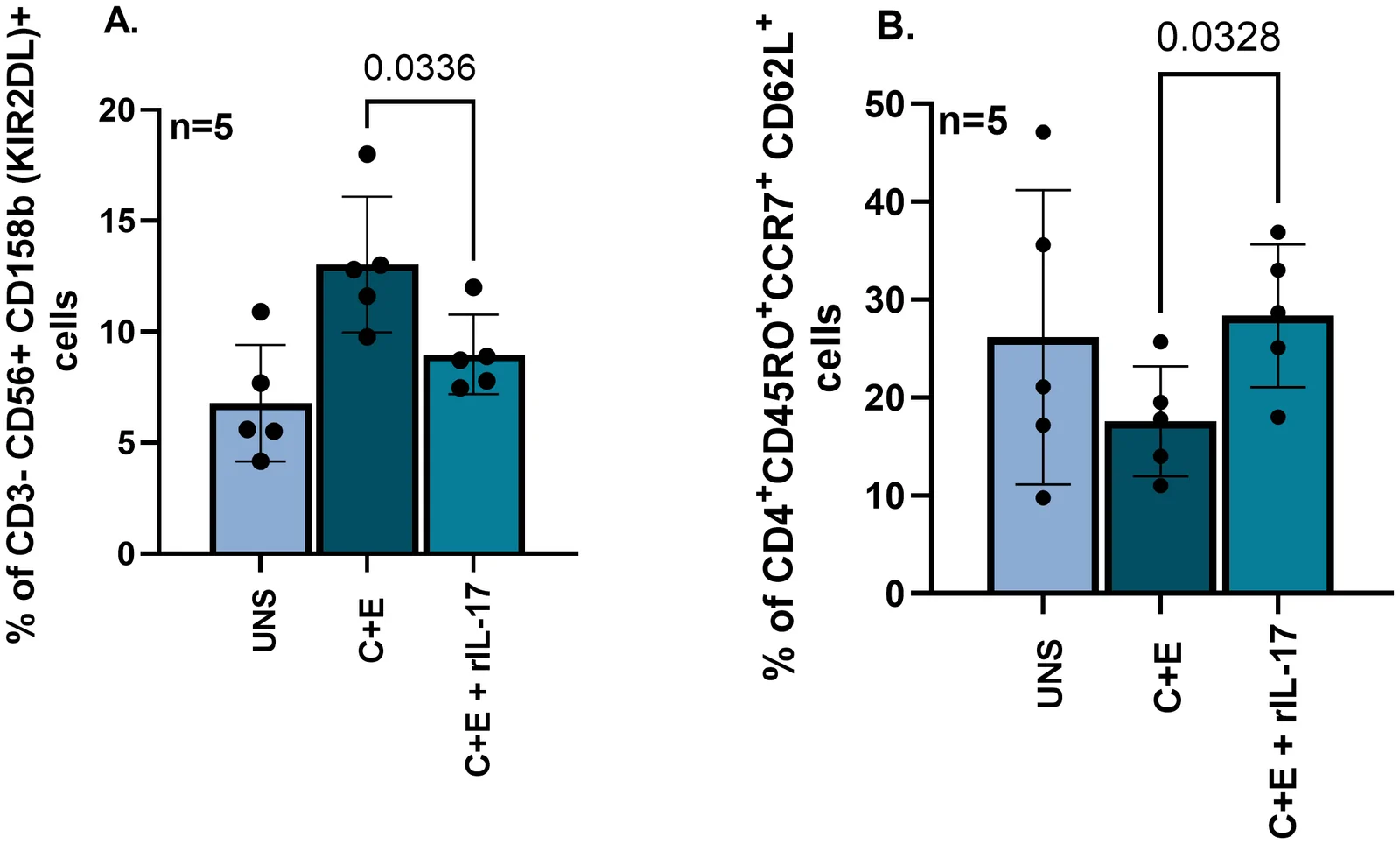
Immune cell populations in CFP-10 and ESAT-6 stimulated PBMC of HIV+LTBI+ children in the presence or absence of recombinant IL-17. PBMCs were isolated from HIV+LTBI+ children and cultured with or without ESAT-6 and CFP-10 (10 μg/mL each). Some ESAT-6 and CFP-10 (10 μg/mL each) stimulated cells were cultured with recombinant IL-17 (10 ng/mL). After 96 hours, immune cell populations were determined by flow cytometry, **(A)** CD3-CD56+CD158b (KIR2DL)+ cells **(B)** CD4+CD45RO+CCR7+CD62L+ cells. The number of donors are shown adjacent to Y-axis. *P* values were determined using one-way ANOVA with Tukey’s multiple-comparison test. Mean values, SEM, and *P* values are shown.

In the above cultured cells, we determined the expression of markers for various immune cell populations. Recombinant IL-17 significantly decreased CD3-CD56+CD158b (KIR2DL)+ (*P* = 0.0336, **Figure 6A**) expressing NK cell population by CFP-10 and ESAT-6 cultured PBMC. In contrast, recombinant IL-17 significantly increased CD4+CD45RO+CCR7+CD62L+ (*P* = 0.0328, **Figure 6B**). expressing T cell population by CFP-10 and ESAT-6 cultured PBMC.

## Discussion

HIV infection alters the balance between the innate and adaptive immune systems, increasing susceptibility to infections by compromising the immune response (20). In this study, we compared immune cell phenotypes, Mtb antigen-specific cytokine production and the factors that reduce the production of the protective cytokine IL-1β by PBMCs from HIV+LTBI+ children and HIV-LTBI+ children. We found that compared with HIV-LTBI+ (healthy LTBI+), HIV+ children with latent TB have significantly fewer CD3-LAG3+CD56+ NK cells, CD8+CD45RA+CD45RO-, CD8+CD45RA+CCR7+ and CD8+CD45RA+CCR7+CXCR3+ cells among their PBMCs. We also found that in response to Mtb antigens, compared with HIV-LTBI+ children, PBMCs from HIV+LTBI+ children produced less IL-1β, IL-17, IFN-γ and MCP-1. Recombinant IL-17 significantly increased the production of IL-1β, MCP-1 and MIP-1α; increased the number of CD4+CD45RO+CCR7+CD62L+ T cells; and significantly decreased the number of CD3-CD56+CD158b (KIR2DL)-expressing NK cells among CFP-10- and ESAT-6-stimulated PBMCs from HIV+LTBI+ children. Our findings demonstrate that defective IL-17 production is associated with reduced secretion of the protective cytokine IL-1β in HIV+LTBI+ children.

In HIV infected adults with LTBI, CD3^-^CD56^+^CD16^+^ lymphocytes are not only significantly low in number but also show impaired function (significantly low expression of cytolytic granules) and reduced cytokine production in response to Mtb antigens (16). The PBMCs of HIV+LTBI+ (HIV+ latent TB) children contained significantly fewer CD3-LAG3+CD56+ NK cells, than those of HIV-LTBI+ (healthy latent TB) children (**Figure 2A**). LAG3 expression on NK cells indicates that they are activated but in an exhausted hyporesponsive state (21). LAG3 expression inversely correlates with the CD4/CD8 ratio and CD4 T-cell count in HIV-infected people (22, 23). The mechanisms underlying the correlation between LAG3 expression and the CD4/CD8 T-cell ratio in HIV patients are not clearly understood (22). Antibody mediated blockade of the interaction between LAG3 and its ligands enhances the function of NK cells by increasing IFN-γ and TNF-α secretion (24).

HIV infection drives premature aging of the T-cell repertoire (25). We found that healthy HIV negative children have high proportions of naive CD8^+^ T cells (CD45RA^+^CD45RO^-^) and few memory cells reflecting limited antigen exposure (**Figures 3A, B**). During infection, CD8+CD45RA+CCR7+ cells are naïve CD8+ T cells that migrate to lymph nodes and secondary lymphoid organs (26). Reduced number of Th1 associated naïve CD8+CD45RA+CD45RO- cells in the blood of HIV+LTBI+ children suggests that they could be migrating from the blood to lymph nodes. During HIV infection CXCR3 expression on CD8 T cells is shown to positively correlate with CD4 counts and negatively with viral loads (27). We observed that these cells are low in HIV+LTBI+ children suggesting that they are unable to migrate into the infected tissues to control viral replication contributing to impaired responses to Mtb.

In response to Mtb antigens, the PBMCs of HIV+LTBI+ children produced low levels of IFN-γ, IL-17A, IL-1β, and MCP-1 compared to the PBMCs of HIV-LTBI+ children (**Figures 4A–D**). IFN-γ is known for its protective role in TB, however, in HIV infected individuals with latent TB, IFN-γ levels were shown to be affected by viral loads and degree of exposure to Mtb (13, 28, 29). We hypothesized that IFN-γ is important to control Mtb infection, but in the context of HIV, it has to be paired with other cytokines. During HIV progression, proinflammatory IL-17A expression decreases but long-term HAART leads to the restoration of IL-17A expression (30). IL-17 plays an important role in vaccine-induced protective immune responses against Mtb (31). IL-17A-deficient mice are susceptible to Mtb infection (32). HIV-1 has been reported to induce IL-1β expression and inflammasome activation in human monocytes (33). Mtb-infected macrophages produce IL-1β (34), which is essential for the restriction of Mtb growth (35). Macrophages express IL-17 receptors, and macrophage-derived IL-1β enhances IL-17 production (36). IL-17A has been shown to drive the production of IL-1β by macrophages (36, 37). HIV infection can reduce IL-17A and IL-1β production in response to TB (38, 39). The cytokine IL-17A is produced mainly by CD4+ helper T cells called Th17 cells (40). However, the specific cellular sources of IL-17A were not determined in this study due to blood sample volume constraints in HIV+ children. Recombinant IL-17A treatment significantly enhanced IL-1β production (**Figure 5A**). IL-17 is produced by Th17 (41, 42), γδ T cell (43) and innate lymphoid cells (ILC3s) (44, 45) during Mtb infection. Our findings demonstrate that optimal IL-17 production is essential for optimal IL-1β secretion by monocyte and macrophage in HIV+LTBI+ children.

IL-17 affects neutrophil chemotaxis, neutrophil expansion, and myeloid and mesenchymal cell functions (46). We found that recombinant IL-17 can significantly increase IL-1β, MCP-1, and MIP-1α production by ESAT-6- and CFP-10-stimulated PBMCs from HIV+LTBI+ children (**Figures 5A–C**). MIP-1α acts by competitively inhibiting viral entry into target cells through binding with CCR5, the HIV coreceptor, which is associated with decreased viral replication and a reduced viral load (47). IL-17 acts through PBMCs to upregulate MIP-1α expression via NF-κB signaling, thereby increasing β-chemokine release in the inflammatory microenvironment. MIP-1α interferes with the HIV binding of CCR5 by decreasing the number of target cells that are permissive to viral infection (47)​​. Mtb infection can lead to chronic immune activation that facilitates HIV entry into CD4+ T cells in the lungs (48). We found that IL-17 enhances MIP-1α production by Mtb stimulated PBMCs in HIV+LTBI+ children. MIP-1α also controls the replication of HIV by blocking viral entry into CD4+ T cells and controlling the exhaustion of T cells (49). In contrast, MCP-1 (CCL2) mainly recruits monocytes/macrophages and is involved in the regulation of inflammatory responses. MCP-1 prevents excessive immune activation, which is an important mechanism for avoiding immune-mediated tissue damage in chronic infections such as HIV (50).​ IL-17 mediates the upregulation of MCP-1 through the Act1/TRAF6/TAK1 signaling cascade (51). MCP-1 upregulation enhances monocyte recruitment and polarization, helping to resolve inflammation while limiting viral spread via immune surveillance (52). Our findings suggest that IL-17 is important for MCP-1-mediated monocyte recruitment for immune surveillance and possibly helps drive a balanced inflammatory response that prevents immune overactivation while supporting pathogen clearance (53).

Recombinant IL-17A significantly increased the expansion of CD4+CD45RO+CCR7+CD62L+ cells in PBMCs from HIV+LTBI+ children stimulated with ESAT-6 and CFP-10 (**Figure 6B**). CD4+CD45RO+ cells represent a memory helper T-cell lineage that has been exposed to antigens, while CCR7 and CD62L guide T-cell recruitment to lymph nodes and secondary lymphoid tissues (54). Our findings demonstrate that IL-17 enhances the expansion of Mtb specific CD4+ central memory T cells (**Figure 6B**). IL-17 plays a protective role against Mtb in lymphoid tissue (32). IL-17 decreased the expansion of CD3-CD56+CD158b (KIR2DL)+ cells in the PBMCs of HIV+LTBI+ children (**Figure 6A**). In viral infections CD158b (KIR2DL)+ cells bind to HLA-C to inhibit NK cell activity (55).

Our finding that recombinant IL-17 restores the production of protective inflammatory mediators (IL-1β, MCP-1, and MIP-1α) and enhances memory like CD4+ T cell expansion highlights IL-17 deficiency is one of the reasons for immune dysfunction in HIV+LTBI+ children. This aligns with recent observations by Mthembu et al. (2024) in the adult population, where a lack of IL-17A producing CD8+ T cells in the lung mucosa was identified as a critical determinant of compromised anti mycobacterial immunity (13). Recombinant IL-17A can partially restore the impaired IL-1β response to Mtb antigens (**Figure 5A**), suggesting IL-17 based therapeutic strategies may help to prevent active TB progression in HIV+ children. However, stronger *in vivo* evidence and preclinical modeling are required. Given the study limitations, our findings therefore highlight a novel pathway of immune dysregulation in HIV+LTBI+ children.

### Study limitations

We acknowledge that our HIV+LTBI+ cohort is relatively small, consisting of 14 participants. Applying strict clinical and radiological diagnostic criteria to confidently exclude active TB and any other opportunistic infections in immunosuppressed children narrowed our inclusion margin. Combined with strict pediatric blood draw volume limits for multi assay PBMC isolation, a small cohort was unavoidable. Nevertheless, the statistical differences identified in our cytokine and cellular profiling remain robust, indicating a pronounced biological effect size that justifies the current sample scale. Future multi-center collaborations will aim to validate these findings on a larger scale.

Statistical limitations: Due to the modest sample size in the HIV+LTBI+ group (n = 14 in functional assays), complex multivariable models integrating concurrent adjustments for age, gender, BMI, CD4 count, and viral load were restricted to prevent model over fitting. To prevent baseline confounding while avoiding over fitting models, the functional assays were conducted on age, gender and BMI matched sub groups to ensure valid comparisons across study groups.

## Data Availability

The original contributions presented in the study are included in the article/**Supplementary Material**. Further inquiries can be directed to the corresponding author.

## Supplementary material

Supplementary Table 1 List of fluorochrome markers used to stain the fresh PBMC from HIV- and HIV+ children.

Supplementary Table 2 QuantiFERON-TB Gold Plus (QFT-Plus) interferon-gamma (IFN-γ) response values across clinical groups.

Supplementary Figure 1 Flow cytometry gating hierarchy for PBMC subset profiling. Representative gating strategy showing the sequential identification of immune cell populations from human PBMCs. After excluding debris (FSC-H vs. SSC-H) and doublets (FSC-H vs. FSC-A), single lymphocytes were gated into distinct subsets: CD3-CD56+ NK cells assessed for KLRG1, NKG2D, LAG3, CD158b, and IL-21R; CD8+ T cells discriminated by CD45RA and CD45RO, with further profiling of CD8+CD45RA+ cells for CCR7, CD62L, CXCR3, and CD69; CD4+ T cells including CD25+FoxP3+ regulatory T cells evaluated for HLADR, PD-1, and CXCR5 expression; and CD14/CD16 myeloid and NK subsets, including CD56+CD16+ cells.

Supplementary Figure 2 Immune cell populations in the freshly isolated PBMC. PBMCs were isolated from HIV-LTBI-, HIV-LTBI+, HIV+LTBI- and HIV+LTBI+ children and were stained with antibodies to monocyte and T cell subsets. Percentages of **(A)** CD14+CD16+ Cells **(B)** CD8+CD45RA+CD45RO+ **(C)** CD8+CD45RA+CCR7+CD69+ (D) CD4+CD25+Foxp3+PD1+ cells were determined by flow cytometry. The number of donors per group are shown above each bar. *P* values were determined using one-way ANOVA with Tukey’s multiple-comparison test. Mean values, SEM, and *P* values are shown.

Supplementary Figure 3 Immune cell populations in the freshly isolated PBMC. PBMCs were isolated from HIV-LTBI-, HIV-LTBI+, HIV+LTBI- and HIV+LTBI+ children were stained with antibodies to monocyte, NK cells and T cell subsets. Percentages of **(A)** CD14-CD16+ **(B)** CD16+CD56+ **(C)** CD3-CD27+CD56+IL-21R+ NK cells **(D)** CD4+ T cells **(E)** CD4+CD25+FoxP3+CXCR5+ (F) CD4+CD25+FoxP3+HLADR+ cells were determined by flow cytometry. The number of donors per group are shown above each bar. *P* values were determined using one-way ANOVA with Tukey’s multiple-comparison test. Mean values, SEM, and *P* values are shown.

Supplementary Figure 4 Cytokine profiles of ESAT-6 and CFP-10 stimulated PBMC. PBMC were isolated from HIV-LTBI-, HIV-LTBI+, HIV+LTBI- and HIV+LTBI+ children and cultured with or without ESAT-6 and CFP-10 (10 μg/mL each). After 96 hours, the culture supernatants were collected, and levels of following cytokines were measured by ELISA. **(A)** IL-10 **(B)** IL-1α **(C)** IL-6 **(D)** IL-22. The number of donors per group are shown above each bar. *P* values were determined using one-way ANOVA with Tukey’s multiple-comparison test. Mean values, SEM, and *P* values are shown.

Supplementary Figure 5 Cytokine and chemokine levels in unstimulated (nil) and mitogen stimulated supernatants of QuantiFERON-TB Gold Plus assay tubes. Unstimulated (nil) and mitogen (PHA) stimulated supernatants from the QuantiFERON-TB Gold Plus assay tubes were collected from HIV-LTBI-, HIV-LTBI+, HIV+LTBI- and HIV+LTBI+ children. Levels of **(A)** IL-1β **(B)** IL-17A **(C)** MCP-1 were determined by ELISA. The number of donors per group are shown above each bar. *P* values were determined using one-way ANOVA with Tukey’s multiple-comparison test. Mean values, SEM, and *P* values are shown.

Supplementary Figure 6 LDH (Lactate Dehydrogenase) cytotoxicity assay in CFP-10 and ESAT-6 stimulated PBMCs. PBMCs were isolated from HIV-LTBI-, HIV-LTBI+, HIV+LTBI- and HIV+LTBI+ children and cultured with or without ESAT-6 and CFP-10 (10 μg/mL each), as described in methods section. After 96 hours, culture supernatants were collected and LDH levels was measured by calorimetry assay. The number of donors per group are shown above each bar. *P* values were determined using one-way ANOVA with Tukey’s multiple-comparison test. Mean values, SEM, and *P* values are shown.
