# Supplementary material for: Defective IL-17 production is associated with reduced IL-1β levels in response to Mycobacterium tuberculosis in HIV+ children with latent tuberculosis infection
Source: Front Immunol. 2026 Sep 9;17:1784204. doi: 10.3389/fimmu.2026.1784204 (PMC13597363; doi:10.3389/fimmu.2026.1784204)
Supplement: Supplementary file 1 [file DataSheet1.pdf]

Supplemental Table 1. List of fluorochrome markers used to stain the fresh PBMC from HIV- and HIV+ children

Panel	Antibody	Fluorochrome	Catalogue number	Manufacturer
**NK cell Memory**	CD3	FITC	555332	BD Biosciences, San Jose, CA, USA
	CD27	PE	555441	BD Biosciences, San Jose, CA, USA
	CCR7	PerCP	353242	BioLegend, San Diego, CA, USA
	PD1	PE/Cy7	561272	BD Biosciences, San Jose, CA, USA
	CD56	APC	555518	BD Biosciences, San Jose, CA, USA
	CD16	APC-H7	560195	BD Biosciences, San Jose, CA, USA
	IL21R	Brilliant Violet 421	565963	BD Biosciences, San Jose, CA, USA
	KLRG1	Brilliant Violet 510	138421	BioLegend, San Diego, CA, USA
**Cytotoxic NK cells**	TIM3	FITC	565568	BD Biosciences, San Jose, CA, USA
	CD226 (DNAM-1)	PE	559789	BD Biosciences, San Jose, CA, USA
	NKG2A	PerCP	747926	BD Biosciences, San Jose, CA, USA
	NKG2D	PE/Cy7	562365	BD Biosciences, San Jose, CA, USA
	CD56	APC	555518	BD Biosciences, San Jose, CA, USA
	CD3	APC-H7	560176	BD Biosciences, San Jose, CA, USA
	CD152	Brilliant Violet 421	562743	BD Biosciences, San Jose, CA, USA
	LAG3	Brilliant Violet 510	569616	BD Biosciences, San Jose, CA, USA
**T cells**	CD4	FITC	566320	BD Biosciences, San Jose, CA, USA
	CD8	PE	555367	BD Biosciences, San Jose, CA, USA
	CD45RA	PerCP	563429	BD Biosciences, San Jose, CA, USA
	CD62L	PE/Cy7	565535	BD Biosciences, San Jose, CA, USA
	CD45RO	APC	559865	BD Biosciences, San Jose, CA, USA
	CD69	APC-H7	560737	BD Biosciences, San Jose, CA, USA
	CXCR3	Brilliant Violet 421	562558	BD Biosciences, San Jose, CA, USA
	CCR7	Brilliant Violet 510	563449	BD Biosciences, San Jose, CA, USA
**Monocytes**	CD11c	FITC	561355	BD Biosciences, San Jose, CA, USA
	CD16	PE	561313	BD Biosciences, San Jose, CA, USA
	CD14	PerCP	325632	BioLegend, San Diego, CA, USA
	HLA-DR	PE/Cy7	335795	BD Biosciences, San Jose, CA, USA
	CD15	APC	551376	BD Biosciences, San Jose, CA, USA
	CD64	APC-H7	561190	BD Biosciences, San Jose, CA, USA
	CCR2	Brilliant Violet 421	564067	BD Biosciences, San Jose, CA, USA
	CD11b	Brilliant Violet 510	301334	BioLegend, San Diego, CA, USA
**NK cells with KIR**	CD3	FITC	555332	BD Biosciences, San Jose, CA, USA
	CD85j	PE	551053	BD Biosciences, San Jose, CA, USA
	CD158b	PerCP	746236	BD Biosciences, San Jose, CA, USA
	CD158	PE/Cy7	339512	BioLegend, San Diego, CA, USA
	CD56	APC	555518	BD Biosciences, San Jose, CA, USA
	TIGIT	Brilliant Violet 421	570375	BD Biosciences, San Jose, CA, USA
	CD158e1	Brilliant Violet 510	742980	BD Biosciences, San Jose, CA, USA
**Regulatory T cells**	CD4	FITC	566320	BD Biosciences, San Jose, CA, USA
	FOXP3	PE	560046	BD Biosciences, San Jose, CA, USA
	HLA-DR	PerCP	552764	BD Biosciences, San Jose, CA, USA
	PD1	PE/Cy7	561272	BD Biosciences, San Jose, CA, USA

	CD25	APC	555434	BD Biosciences, San Jose, CA, USA
	CD45RA	APC-H7	560674	BD Biosciences, San Jose, CA, USA
	CD152	Brilliant Violet 421	562743	BD Biosciences, San Jose, CA, USA
	CXCR5	Brilliant Violet 510	563105	BD Biosciences, San Jose, CA, USA
