# Supplementary material for: Defective IL-17 production is associated with reduced IL-1β levels in response to Mycobacterium tuberculosis in HIV+ children with latent tuberculosis infection
Source: Front Immunol. 2026 Sep 9;17:1784204. doi: 10.3389/fimmu.2026.1784204 (PMC13597363; doi:10.3389/fimmu.2026.1784204)
Supplement: Supplementary file 2 [file DataSheet2.pdf]

Supplementary Table 2. QuantiFERON-TB Gold Plus (QFT-Plus) interferon-gamma (IFN-γ) response values across clinical groups.

Group	Unstimulated or Nil (IU/mL)	TB1 (IU/mL)	TB2 (IU/mL)	Mitogen (IU/mL)	p-value (Mitogen)
HIV-LTBI- (n=125)	0.18 ± 0.92	0.27 ± 1.01	0.26 ± 1.01	4.89 ± 2.25	0.256 (ns)
HIV-LTBI+ (n=25)	0.48 ± 0.72	1.81 ± 2.09	1.78 ± 1.96	4.83 ± 2.32	
HIV+LTBI- (n=38)	0.10 ± 0.08	0.14 ± 0.12	0.16 ± 0.15	4.06 ± 2.28	
HIV+LTBI+ (n=14)	0.34 ± 0.37	1.05 ± 1.77	2.02 ± 2.23	5.02 ± 2.66	

Values represent interferon-gamma (IFN-γ) concentration in international units per milliliter (IU/mL) expressed as Mean ± SD. Abbreviations: HIV, Human Immunodeficiency Virus; LTBI, Latent Tuberculosis Infection; Nil, negative control tube/unstimulated (background IFN-γ); TB1, Tuberculosis Antigen 1 (stimulates CD4+ T-cells); TB2, Tuberculosis Antigen 2 (stimulates both CD4+ and CD8+ T-cells); Mitogen, positive control tube; SD, Standard Deviation; ns, not statistically significant ($p > 0.05$); Statistical analysis between groups for Mitogen responses was performed using one-way Analysis of Variance (ANOVA); $p = 0.256$ (ns, $p > 0.05$), indicates no significant difference in mitogen induced IFN-γ secretion across the study groups.
